# Impact of crop commercialization on multidimensional poverty in rural Ethiopia: propensity score approach

**DOI:** 10.3389/fpubh.2024.1412670

**Published:** 2025-01-09

**Authors:** Anteneh Mulugeta Eyasu, Temesgen Zewotir, Zelalem G. Dessie

**Affiliations:** ^1^Department of Statistics, College of Science, Bahir Dar University, Bahir Dar, Ethiopia; ^2^Department of Agricultural Economics, College of Agriculture and Environmental Science, Bahir Dar University, Bahir Dar, Ethiopia; ^3^School of Mathematics, Statistics and Computer Science, University of KwaZulu-Natal, Durban, South Africa

**Keywords:** rural multidimensional poverty, crop commercialization, Alkire and Foster method, propensity score, generalized linear mixed-effects model

## Abstract

**Introduction:**

Reducing poverty through crop commercialization is one of the antipoverty efforts that helps promote health. This study explored the prevalence and the causal relationship between crop commercialization and rural Ethiopian households’ multidimensional poverty using multilevel data.

**Methods:**

The study uses data from the most recent nationally representative Ethiopian socioeconomic survey 2018/19 to calculate the rural multidimensional poverty index using the Alkire and Foster technique. The data show 2,714 rural households nested in 59 administrative zones of Ethiopia. Based on several parameters (nutrition and health, education, living standards, rural livelihoods and resources, and risk), the investigation looks into the multidimensional poverty levels of Ethiopian rural households and how they differ across Ethiopian administrative zones.

**Results:**

The results indicate that 47.8% of the rural households of Ethiopians were multidimensionally poor in several dimensions; nutrition and health, education, living standards, rural livelihoods and resources, and risk. The living standard dimension is most deprivation-prone for the rural, multidimensional poor households. In addition, multidimensional poverty is more prevalent in Somali and Afar region rural areas. The best linear unbiased prediction estimates of multidimensional poverty vary substantially across Ethiopia’s administrative zones. Specifically, the top poorest performing administrative zones concerning the likelihood of being multidimensional poor among rural households were Shebelle, Zone 2, Zone 3, Zone 4, and Konso special woreda.

**Conclusion:**

The results of the generalized linear mixed-effects model show that crop-commercialized households have reduced the odds of being multidimensionally poorer than those who did not. This study recommends policymakers focus on rural mumyltidimensional poverty reduction strategies.

## 1 Introduction

Poverty is usually determined by the level of income, consumption expenditure, and sufficient basic resources to maintain sustainable livelihoods. It is also a display of educational inaccessibility, hunger and malnutrition, social unfairness, and limited access to other basic needs (1). The monetary approach (household income or consumption expenditure) is a good proxy for measuring poverty because it shows the welfare level of the household. However, they do not measure deprivations in different dimensions aside from income, hence not providing sufficient policy guidance in these dimensions (2, 3). It goes beyond just monetary terms to include other dimensions of deprivation, such as adult education level, as well as the availability of sufficient electric power and hygiene (4). The increasing use of poverty measurement in terms of welfare outcome indicators reflects housing quality, overcrowding, and access to essential services such as water, sanitation, healthcare, and education (5). A multidimensional poverty measure is based on the capability approach and helps policymakers identify and track the poor who are deprived in several areas, including money, health, education, and living standards (6). It can also be defined as the failure to reach a predetermined level in several aspects of a person’s wellbeing (7). Thus, rather than focusing on changes in inputs like income or consumption, poverty is assessed in terms of gains in outcomes, such as human development or maintaining a nutritious diet (8). Hence, several researchers have suggested the measurement of poverty indicators should go beyond expenditure or income to a multidimensional approach for showing household deprivations in various dimensions of poverty (5, 9–11).

The primary goal of national and international development organizations, as well as the first Sustainable Development Goal (SDG) of the United Nations, is eradicating poverty worldwide in all its dimensions (12–14). The existing evidence indicates that most of the world’s monetary poor (81.3 percent) live in rural areas, and according to the global multidimensional poverty index introduced, 85 percent of poor people live in rural areas. Specifically, in South Asia and sub-Saharan Africa, the percentage of multidimensionally impoverished people is much higher (83%) (4, 15). Hence, the world’s multidimensional poor live predominately in rural and remote areas that require an accurate and specifically tailored rural multidimensional measure to capture the full spectrum of deprivations faced by rural communities (16, 17). Even though several studies of poverty focused on a multidimensional approach (18–21), they used only three dimensions (education, health, and living standards) and ten indicators (11, 18, 19), others used four dimensions and 14 indicators (20, 21) and their study covers limited areas in Ethiopia (19–24). Thus, to address this dimensional and indicators limitation of poverty and to better reflect the rural household livelihoods, following the recent work of FAO and OPHI (16) and Vollmer et al. (17), this study used multidimensional poverty measurement for rural households by including sixteen indicators across five dimensions: nutrition and health, education, living standards, rural livelihoods and resources, and risk. According to the recommendations of FAO and OPHI (16) and Vollmer et al. (17), we have added two crucial dimensions that rural households are vulnerable and exposed to rural livelihoods and resources, and risks. Unlike other poverty measures, this rural multidimensional poverty measure includes innovative indicators on rural social protection, extension service, agricultural assets adequacy, and exposure to risk and coping mechanisms.

Out of 112 countries in 2024, the global Multidimensional Poverty Index reported that 1.1 billion people live in multidimensional poverty. Nearly half (48.2 percent) of these poor people live in Sub-Saharan Africa. With approximately 86 million poor people, Ethiopia ranks as the first poorest country in Africa as well as it is the third poorest country in the world, next to India and Pakistan. A large proportion of all poor people lack adequate sanitation, housing, and cooking fuel. Moreover, over half of all poor people live with a person who is undernourished in their household. Approximately half of all poor people lack electricity, and over half live in a household where no one has completed 6 years of schooling. Nearly half of all poor people do not have an improved source of drinking water. Approximately 482 million poor people live in households where one or more children are out of school (25). Approximately 80 percent of Ethiopia’s rural population lives in multidimensionally impoverished households. This indicates that 42.6% of all deprivations experienced by the multidimensionally poor in rural Ethiopia would take place if every member of these rural societies were completely deficient in every indicator (16). Ethiopia had a decrease in multidimensional poverty from 0.491 in 2011 to 0.436 in 2016 to 0.367 in 2019, as well as a decrease in the incidence of poverty from 83.5 percent to 77.4 percent to 68.8 percent. This decline was primarily caused by a decline in the proportion of the impoverished who lack years of education, which was followed by declines in the proportions of those who lack assets, housing, cooking fuel, electricity, and clean water to drink (26).

Global hunger and food insecurity are critical challenges that affect millions of people worldwide. Approximately 733 million people faced hunger in 2023 globally (one in eleven people) and one in five people (20.4%) in Africa. In 2023, approximately 2.33 billion people globally faced moderate or severe food insecurity. This challenge also continues in Africa (58%). Chronic hunger can lead to malnutrition and even starvation, posing a severe threat to health and child development. The root causes of this global food crisis are poverty, conflict, climate change, and economic instability. Poverty is one of the leading causes of food insecurity, and it limits people’s ability to afford food and their access to education and healthcare. Climate changes such as droughts and floods pose a major and growing threat to global food security by reducing crop and animal yields. It also impacts the livelihoods of farmers and the health of vulnerable communities. Efforts to address global hunger and food insecurity problems involve a combination of strategies, including improving food production and distribution systems, promoting sustainable agriculture, reducing poverty, enhancing social safety nets, and addressing the underlying causes of these issues, such as conflict and climate change (27). Despite Ethiopia has work to achieve the Sustainable Development Goal of zero hunger by 2030, with a score of 26.2 in the 2024 global hunger index, it has a serious level of hunger and ranks 102nd out of the 127 countries (28). Food insecurity and malnutrition are still a major concern in Ethiopia due to recurrent conflict, droughts, diseases, and inflation (29). Ethiopia continues to have extremely high levels of poverty due to conflict, the COVID-19 pandemic, the invasion of locusts, and international conditions that drive up food prices, poor urban planning, low educational attainment, weak institutions, heavy resource exploitation, frequent extreme weather events, and the long-term effects of climate change that threaten livelihoods in agriculture and pastoralists as well as food security (4, 26, 30). Hence, because rural livelihoods are dependent on rain-fed agriculture, they are particularly vulnerable to many risks and shocks that could cause them to fall back into poverty (31–33).

To identify the poor, comprehend their living circumstances and the unique barriers preventing them from escaping poverty, and ultimately create integrated agriculture-related policies to end poverty, it is helpful to quantify rural poverty. In Ethiopia, various strategies have helped in reducing poverty; in 2020/2021, the country’s economy grew by 6.3%, and agriculture accounted for 37.57 percent of the GDP, followed by the services sector at 36.25 percent and industry at 21.85 percent. Thus, more than 70% of the population works in agriculture, its contribution to growth is slightly improved, and it has a great role in poverty reduction. There are also main pro-poor government initiatives such as the Productive Safety Net Programme (PSNP) and the Multisectoral Woreda Transformation Program. The percentage of people living in rural areas below the national monetary poverty level dropped from 30% in 2011 to 26% in 2016 (34, 35). The commercialization of agriculture can be a major factor in rural development and the fight against poverty. It is also a major force behind structural change. The level of a farm household’s market connectivity is known as commercialization (36, 37). An empirical study using quantile regressions in Kenya showed commercialization of agriculture has a favorable impact on income poverty and multidimensional poverty (18). Reducing poverty and enhancing the welfare of rural farm households in Ethiopia can be achieved through the commercialization of smallholder agriculture. A statistically significant decrease in the impact of crop commercialization on multidimensional poverty and susceptibility to it was observed in Ethiopian Teff-based mixed farming areas, according to the research conducted using the instrumental variable Tobit. Further evidence from the data suggests that commercialization is a crucial pro-poor growth strategy for lowering agricultural households’ poverty in rural Ethiopia (24). Even though a huge investment was made in Ethiopia’s Growth and Transformation Plan I (GTP I from 2010/11 to 2014/15) and II (GTP II from 2015/16 to 2019/2020) for smallholder commercialization as a means of agricultural sector transformation to help farmers gain higher incomes and promote rural development, commercialization is not promoted sufficiently. Farmers are still practicing subsistence farming and are ill-equipped to handle the shocks and strains brought on by rising prices and climate change (38). Reducing poverty through crop commercialization is one of the antipoverty efforts that helps promote health. Thus, there is a need for an extensive study to identify the extent and impact of crop commercialization on the multidimensional poverty experienced by rural households.

Agricultural commercialization has been found to have various effects on different aspects of rural household’s welfare. It is a pressing issue in African countries (Malawi, Tanzania, and Uganda) and enhances trade and efficiency, leading to economic growth and welfare improvement (39). Other studies in Kenya by Ogutuet al. (40) remarked that commercialization significantly improves food security and dietary quality. A higher level of commercialization contributes to higher incomes by improving their consumption of purchased food and increasing nutrient (calorie, zinc, and iron) consumption. The previous studies in rural Vietnam have demonstrated that households who commercialize rice were better off in terms of asset accumulation and income. They also noted that agricultural commercialization increases household consumption (37). The commercialization of food crops (bananas and legumes) has a positive significant effect on dietary diversity and farm income of rural households in Rwanda and the Democratic Republic of Congo (41). The studies in Chinese rural households by Zheng and Ma (42) also found that households with higher agricultural commercialization rates are significantly increasing rural households’ dietary diversity and are less vulnerable to poverty. The previous studies in rural Vietnam showed that agricultural commercialization reduces multidimensional poverty (43). The study in maize-producing regions of Ethiopia (Oromia, the Southern Nations, Nationalities and Peoples, and the Benishangul-Gumuz) by Geffersa and Tabe-Ojong (22) found that a positive association between smallholder maize farmers’ commercialization and household income which translates to wealth endowments through asset ownership and accumulation. They also show that commercialization is associated with poverty reduction by reducing the prevalence of income poverty. Moreover, the study in selected regions (Tigray, Amhara, Oromia, and southern nations, nationalities peoples) of Ethiopia found that crop commercialization is positively correlated with per capita income (23).

However, most of the prior studies analyzing poverty effects of commercialization considered indicators such as per capita income or per capita consumption (22, 23, 37–42, 44). Even though this monetary approach to measuring poverty is widely used, it does not fully reflect the multidimensional nature of poverty (18, 24, 43). Hence, this study contributes to the existing literature by analyzing the impact of crop commercialization on multidimensional poverty in the rural Ethiopia context. Unlike most previous studies (22–24, 38), this study utilized the nationally representative and recent data of rural households in Ethiopia. This enables us to compare multidimensional poverty across administrative zones, regions, genders, and crop commercialization groups. In addition, no previous studies incorporate the heterogeneity issues that arise in estimating the impact of crop commercialization on multidimensional poverty across administrative zones of Ethiopia.

Numerous fields (patients in hospitals, people in geographic locations, and students in schools) frequently use clustered data structures. To the degree that the random slope variance and intra-class correlation (ICC) departs from zero, the single-level model that ignores clustering would produce estimates of propensity scores inside single clusters that are progressively biased. Utilizing random effects models to estimate the propensity score has the advantage of mitigating the bias resulting from unmeasured cluster-level variables in propensity score computation techniques (45). The estimation of the propensity score must take into account at the individual and group levels impacts on the likelihood of receiving a treatment assignment in designs for multilevel studies where people within groups are exposed to a treatment (45–49). Hence, this study’s data exhibit a complex dependence structure. Households are nested within administrative zones of Ethiopia, and adjacent households are considered to be geographically correlated. In addition, the observations from households within a zone are likely to be impacted by zone-level policies. The first step in developing effective solutions to reduce poverty is determining the extent and variation of multidimensional rural poverty across Ethiopia’s administrative zones. Hence, by utilizing a generalized linear mixed-effects model with weights from propensity score techniques, this study examines the prevalence and the causal impacts of crop commercialization on the multidimensional poverty of rural households by accounting for the differences in administrative zones in Ethiopia. We considered four main research questions in this study: (1) Which dimensions and indicators of poverty are deprived? (2) What are the potential confounding risk factors of crop commercialization? (3) Does crop commercialization contribute to reducing rural multidimensional poverty? and (4) Which administrative zone ranks best and worst performance in multidimensional poverty reduction?

## 2 Methods

### 2.1 Data

This study used a recent fourth round of nationally representative Ethiopia socioeconomic survey (ESS 2018/19) data; it is not a follow-up to previous ESS waves. Specifically, this study used the rural category of ESS data. These are surveys conducted by the Ethiopian Central Statistical Agency (CSA) and the World Bank’s Living Standards Measurement Study (LSMS). This study sample of rural households was selected from the regions of Amhara, Tigray, SNNP, Oromia, Afar, Somali, Gambella, Benishangul Gumuz, Harari, and Dire Dawa, and a total of 59 administrative zones. This study included a sample of 2,714 households living in rural areas. This nationally representative dataset includes important information about income, expenditure, occupation, demographic aspects, health and education, production activities, asset ownership, agricultural production, and self-reported information on shocks.

The main reasons for this study selected the baseline ESS 2018/19 dataset. First, the ESS wave 4 revised the previous survey instruments, and the changes focused on module updates, guaranteeing that the survey information generated will be in line with the sustainable development goals such as clean water, sanitation, hygiene, labor statistics, and household consumption expenditure survey. Second, to include modules to enhance the quality and accessibility of distinct, disaggregated household data, enabling the tracking of SDG indicators on ownership, usage rights, and decisions made regarding specific material and financial assets. Third, in contrast to other ESS waves, ESS wave 4 is inclusive of Ethiopia’s regions, both rural and urban (50). Hence, the revision of this recent data includes indicators that make it suitable for meaningful measures of rural households’ multidimensional poverty from the same survey in Ethiopia.

### 2.2 Constructing the multidimensional poverty measures

One important worldwide tool for calculating multidimensional poverty is the Global Multidimensional Poverty Index (MPI) which helps to assess how countries meet the SDGs, which is the first goal focused on eliminating poverty in all its manifestations and dimensions. The global MPI aims to address the interconnected deprivations that impoverished people experience to give the most comparable measure feasible for cross-national studies and pertinent data for policies that eliminate poverty (51). The first step in the global MPI is creating a deprivation profile for each household and individual within it. This profile tracks ten indicators that are grouped into three dimensions: living standards (electricity, sanitation, drinking water, housing, cooking fuel, and assets), health (nutrition and child mortality), and education (school attendance and years of schooling). Every dimension has the same weight, and every indicator within a certain dimension has the same weight (11). Multidimensionally poor people are those who are lacking in at least one-third of the weighted indices (16). Hence, this study is based on the Rural MPI by changing some of the global MPI measure’s characteristics and including dimensions and indicators that can more accurately represent rural characteristics, particularly the unique characteristics of rural livelihoods and exposure to possible shocks.

This study employs the Alkire–Foster (AF) methodology for the ESS 2018/19. This method is a way of measuring multidimensional poverty. The Foster–Greer–Thorbecke (FGT) One-dimensional poverty class of metrics is extended to a multidimensional environment by the Alkire–Foster technique. It uses a counting identification approach, which is based on the FGT metrics of poverty (52). A household is considered poor if it exhibits deficiencies in a minimum of k indicators, where k can range from 1 to the overall count of indications included in the study. Based on the chosen deprivation cutoff for each indicator, they employ a two-stage cutoff method, wherein initially, it is essential to determine whether or not each household is deprived in each indicator. Step two involves choosing a value for k and classifying as multidimensionally poor every household who is deficient in k or more indices. The multidimensional index of poverty is generated by this method. Its two components are the average proportion of deficiencies experienced by those living in poverty, or the average intensity of poverty, and the headcount of multidimensional poverty, additionally recognized as incidence. Essentially, the adjusted multidimensional headcount ratio ($M_{0}$) is the product of the incidence (H) and the intensity (A) (11).

The local context and data availability are taken into consideration in the works on multidimensional poverty metrics (10, 11, 16), and we selected five dimensions (nutrition and health, education, living standards, rural livelihoods and resources, and risk) and sixteen indicators of rural multidimensional poverty index (MPI) in Ethiopia (Table 1). We dispersed the weights equally within each dimension according to the number of indicators taken into consideration, and we applied equal weights across all dimensions. Each indicator compares a household’s achievements to a set of deprivation cutoffs to determine whether or not the household is considered deprived. The score of multidimensional deprivation profiles for each household is measured by the Alkire–Foster dual-cutoff counting approach (11), and it categorizes a household as multidimensionally poor if the weighted deprivation score is equal to or greater than 0.333 (16). Finally, stata package mpitb is used to estimate and analyze the multidimensional poverty indices (53, 54).

**Table 1:** Dimensions, indicators, deprivation cutoffs, and weights of the Rural MPI.

Dimension	Indicator	Deprived if	Weight
Nutrition and Health	Child malnutrition	At least one child (aged 6 to 60 months) who lives in the family is stunted and/or underweight	1/10
	Health	No family members use health insurance services	1/10
Education	Years of schooling	No household member aged 13 years or older has finished 6 years of education	1/10
	School attendance	At least one person living in the household who is old enough to finish class 8 does not go to school (all children between the ages of 7 and 15 years)	1/10
Living standards	Electricity	There is no solar energy or electricity in the household	1/30
	Improved sanitation	The sanitary facilities at the home are not upgraded (according to SDG guidelines)	1/30
	Drinking water	Safe drinking water is either unavailable to the household or must be walked at least 30 min each way from the residence	1/30
	Housing	The house is composed of inadequate or unsuitable natural materials, and its roof, walls, and floor are all in poor condition	1/30
	cooking fuel	The household uses charcoal, wood, or manure for cooking	1/30
	Assets	The household does not own more than one of the following assets; television, radio, telephone/mobile phone, refrigerator, bicycle, motorbike, or oxcart, and does not own a vehicle.	1/30
Rural livelihoods and resources	Child labor	A minimum of one member of the household who is younger than 11 years old works in agriculture	1/20
	Extension services	There is no extension service available to any member of the household	1/20
	Agricultural assets adequacy	In terms of cumulative distribution, the land operated by households is situated in the lower 40%	1/20
	Social protection	Members of a household getting government social assistance pension payments, or social protection (iddir)	1/20
Risk	Credit denial	If the household’s loan applications were denied on all occasions	1/10
	Exposure to risk and coping mechanisms	The family experienced covariate shocks, but they employed informal and insufficient coping mechanisms, such as selling possessions, altering their diet, and taking on more employment	1/10

Modified from FAO and OPHI (16).

Data from the fourth wave of the ESS for 2018/19 are used to compute the Rural MPI. This study uses nutrition and health as the first dimension with indicators of child malnutrition and health. Using the anthropometric data on children that are currently available, malnutrition in children is calculated. Children under the age of five are classified as malnourished if their *z*-score for either height-for-age (stunting) or weight-for-age (underweight) is less than two standard deviations from the reference population’s median. The health indicator is the use of health insurance; the household is deprived when there are no family members who utilize health insurance (16, 21, 55). This study used education as the second dimension. The years of schooling and school attendance were used as indications for constructing the education dimension. 13 years of age or older is the minimum age for schooling as the indicator characterizes as deprived anyone with less than 6 years of education. Likewise, anyone studying in the eighth grade or lower, which comprises all children aged from seven to fifteen, is required to attend school (16).

To categorize rural households as deprived or not, the living standards dimension which comprises electricity, better sanitation, drinking water, housing, cooking fuel, and asset indicators is crucial. Sawdust, dung/manure, crop residue, gathered firewood, and charcoal are not considered clean cooking fuels in Ethiopia. The traditional latrines without a slab and shared or non-existent toilet facilities are included in the non-improved sanitation. According to (56), surface water from lakes or rivers, unprotected dug wells, unprotected springs, tanker trucks, piped water from a kiosk or merchant, and carts with small tanks or drums are among the sources of non-safe drinking water. There is no solar energy or electricity in the home. In addition, bamboo, wood, mud, and plastic canvas make up the roofs, while dung and mud make up the flooring and walls of the houses. Computers are not taken into account in the asset indicator when listing the possessions of households (16, 57).

The variables child labor, extension services, agricultural assets adequacy, and social protection were used as indicators for constructing the dimension of rural livelihoods and resources. Child labor has an age threshold of 11 years according to the International Labour Organization, 1973. Furthermore, if a household participated in an extension program or received any advisory services, that information was used to generate Ethiopia’s extension services indicator. Adequate agricultural assets are those that are operated on household land that is located in the lowest 40 percent of the cumulative distribution. The government’s social protection indicator, or iddir, represents the members of a family who get social assistance or a pension. Iddir is a mutual aid funeral organization that helps its members get by socially when a family member passes away. Any member of iddir has the right to use the funds needed to plan and finance a funeral, as well as to assist the family of the member throughout the grieving process (16). Lastly, the risk dimension consists of two indicators credit refusal, and exposure to risk and coping mechanisms. Droughts, floods, intense rains that hinder work, crop damage, changes in food prices, increases in the cost of agricultural inputs, livestock losses, fires, and displacement are examples of covariate shocks in Ethiopia. Some examples of non-formal/inadequate coping mechanisms in the indicator of risk exposure and coping strategies are selling household assets, altering dietary habits, and increasing work hours (16).

This study presented the application of the rural multidimensional poverty index to the Ethiopian context. Due to data availability constraints and limited information, two indicators in the original rural multidimensional poverty index were removed. Hence, this study’s rural multidimensional poverty index included sixteen indicators across five dimensions: nutrition & health, education, living standards, rural livelihoods & resources, and risk. Finally, given the mentioned data limitations, the weights of the indicators within different dimensions were rescaled, as shown in Table 1 following the work of FAO and OPHI (16), while maintaining equal weights across dimensions. The weights were redistributed equally within each dimension based on the number of indicators considered (17, 26).

### 2.3 Crop commercialization index measures

There is no standard definition and measurement of the agricultural commercialization concept. Even though some researchers have considered agricultural commercialization as the growing of cash crops, others have defined agricultural commercialization as not limited to cash crops only as some proportions of food crops are sold for cash, and some proportions of cash crops are consumed at home for example in case of groundnuts in West Africa where a large proportion of groundnuts produced are consumed at home though it is considered as a market-oriented commodity (58). Agricultural commercialization is not only the selling of output but also includes product choice and input use decisions that are based on the profit maximization principle (36, 59). It is central to a structural transformation of agricultural production from growing crops for home consumption to growing some or all crops for sale, and this may facilitate specialization, technology adoption, and greater access to markets and roads that enable farmers to have higher yields and earn cash income. This plays a critical role in improving rural farm household welfare (such as consumption, nutrition, food security, and hunger), economic growth, and sustainable rural development (39, 58, 60, 61). The empirical evidence has also pointed out that agricultural commercialization has an important significant effect on achieving farm household food security, moving out of poverty, increasing income, asset growth, and diet quality improvement (40, 41, 43, 58). The production of marketable surplus staple food crops is usually the most common initial form of commercialization among smallholder farmers (59). Commercialization can potentially create markets for both inputs and outputs, thus pulling investment in rural areas to provide ease of access to goods and services for smallholder farmers and population needs (36).

The household crop commercialization index is defined as a ratio of the gross value of all crop sales per household per year to the gross value of all crop production (62, 63). Most empirical studies adapt it to measure the commercialization of single or some crops (22, 41, 64) and all crops (23, 37, 39, 40, 43, 60, 65). Based on the widely used in the literature and to avoid the potential bias raised when focusing on only one crop (42), hence, this study considered all produced crops by rural households for measuring the household crop commercialization index as the ratio of the total value of all crop output sold to the total value of all crops produced for the production year. This method is used as a standard and more comprehensive approach for measuring household crop commercialization index and to analyze the causal relationship between rural household crop commercialization and multidimensional poverty among administrative zones of Ethiopia. The household crop output market involvement in annual crops is expressed as a percentage of crop sale value to crop production value overall; this is known as the crop commercialization index, or CCI (58, 66, 67) specified as in Equation 1.


(1) $$CCI_{i}=\frac{\sum_{k=1}^{k}{\bar{p}}_{k}s_{ik}}{\sum_{k=1}^{k}{\bar{p}}_{k}Q_{ik}}*100\;$$

Where $s_{ik}$ is the quantity of output *k* sold by household *i* evaluated at an average region level price ${\bar{p}}_{k}$ and $Q_{ik}$ is the total quantity of output *k* produced by household *i*.

The index measures the extent to which household crop production is market-oriented. A value of zero for the CCI shows a completely subsistence-oriented household. The households’ crop commercialization index greater than zero is assigned as treated (crop commercialized household) otherwise a control group.

### 2.4 Propensity score analysis for multilevel data

The propensity score analysis for causal inference involves the following key steps (48): First, assessment of critical covariates that could be considered as potential confounders of crop commercialization based on theoretical or empirical importance. Second, estimate the propensity score by generalized linear mixed-effects model. Third, conditioning on the propensity score methods, such as inverse probability treatment weighting and optimal full matching. Fourth, check covariate balance with metrics such as the absolute standardized mean difference. Researchers may repeat from second to fourth steps to re-estimate the propensity scores using a propensity score model until adequate balance is achieved. The final step in propensity score analysis is an estimation of the treatment effect.

In this study, we estimated the average treatment effect (ATE) using propensity score (PS) methods with multilevel data, that is, every household *i* in cluster *j* is non-randomly assigned to either the control group (***t*** = 0) or treatment group (***t*** = 1). Bannor and Melkamu (68) defines the ATE as $E\left(y_{ij}^{t}\right)-E\left(y_{ij}^{c}\right)$ where $E\left(y_{ij}^{t}\right)$ represents the expected value of the outcome for every individual in the treatment condition, and $E\left(y_{ij}^{c}\right)$ is the expected value of the outcome for every individual in the control condition (49, 69, 70). According to (71), treatment assignment must be unconfounded or strongly ignorable, meaning that prospective outcomes must be apart from the prescribed course of treatment conditioned on covariates and cluster effects $\left(\;\left[y_{ij}^{t}\mathrm{,|,}y_{ij}^{c}⊥t_{ij}\mathrm{,|,}X_{ij}\mathrm{,|,}u_{j}\right]\right).$ For clustered data, even if we collect a sufficiently comprehensive set of pre-treatment variables that influence the treatment and outcome, unmeasured cluster effects $\left(u_{j}\right)$ that are connected to the outcome and the treatment could exist. Therefore, if the cluster effects $\left(u_{j}\right)$ are seen, the average of the possible outcomes can be determined. Considering that the cluster effects are never noticed, so to identify the causal parameter, we make the confounder at the unit level $X_{ij}$, and unreported cluster-level confounding $u_{0j}$ is independent. Strong ignorability for ATE estimation involves both $E\left(y_{ij}^{t}\mathrm{|,,}t_{ij}=1\mathrm{|,,}X_{ij}\mathrm{|,,}u_{j}\right)=E\left(y_{ij}^{t}\mathrm{|,,}t_{ij}=0\mathrm{|,,}X_{ij}\mathrm{|,,}u_{j}\;\right)$ and $E\left(y_{ij}^{C}\mathrm{|,,}t_{ij}=0\mathrm{|,,}X_{ij}\mathrm{|,,}u_{j}\right)=E\left(y_{ij}^{C}\mathrm{|,,}t_{ij}=1\mathrm{|,,}X_{ij}\mathrm{|,,}u_{j}\;\right)$ (49, 72, 73).

Under the ATE, a portion of the propensity score distribution where values for individuals who have received treatment and those who have not is known as the overlap of PS distributions, or enough common support. The stable unit treatment value assumption, or SUTVA, stipulates that study members’ prospective outcomes must be unaffected by the other members’ treatment status and the assignment method. Because members of the same cluster might influence each other’s possible outcomes through interpersonal communication and resource sharing, multilevel data may result in SUTVA breaches. We employed PS techniques in this study with the assumption that SUTVA holds (49, 73, 74).

The multilevel models for the estimation of the treatment effect were implemented with inverse probability treatment weighting and optimal full matching propensity score techniques. Random effects are incorporated into the propensity score model in the propensity score approaches for hierarchical data. They demonstrate how unmeasured heterogeneity resulting from the exclusion of cluster-level confounders in propensity score analysis can be captured by random effects (45, 75). When using a multilevel study design, selection bias caused by clustering may be reduced by taking cluster effects into account in the PS model or the outcome model. However, combining the two approaches will result in the greatest reduction of bias (47, 49, 76). The propensity score for multilevel data using the suggested multilevel logistic regression with random effects is $e\;\left(X_{ij},u_{0j}\right)=P\;\left(t_{ij}=1\mathrm{|,}X_{ij}\mathrm{|,}u_{0j}\right)$ (48).

#### 2.4.1 Propensity score model for multilevel data

One major challenge in evaluating the multilevel data is estimating the propensity scores for the entire sample using a fixed-effect logistic regression model. This is because confounders’ impact on treatment choice might differ throughout clusters. For estimating the propensity score, we examine the possibility of modeling this variance using a random intercept in a multilevel logistic regression model, which is equivalent to a generalized linear mixed-effects model (46, 47, 49, 77):


(2) $$g\left(E\left(t|u_{0j}\right)\right)=X\beta +u_{oj}$$

where ***t*** is the (*n × 1*) vector of treatment (crop commercialization), X denotes the n×p matrix of fixed effect covariates, β is the p×1 unknown vector of parameters for the fixed effects, $u_{0j}$ denotes the random intercept of administrative zone *j* by variance τ, and g(.) denotes the link function, in this case, the logit link function for binomial distributed treatment.

#### 2.4.2 Propensity score methods for multilevel data

This study focused on the application of propensity score methods for estimating treatment effects with multilevel data obtained from observational studies. Propensity score methods are a group of strategies that aim to reduce selection bias by balancing differences between treated and untreated individuals on observed covariates. This study considered the propensity score methods, inverse probability treatment weighing, and optimal full matching to estimate treatment effects with multilevel models through the creation of weights, and estimation of weighted models with the multilevel pseudo-maximum likelihood estimation method (78).

The inverse probability of treatment weighting (IPTW) is the propensity score weighting technique that directly uses the estimated propensity scores to adjust for confounding bias. For estimating the ATE, propensity score weights or inverse probability of treatment weights (IPTW) to multilevel settings are given by $w_{ij}=\frac{1}{{\hat{e}}_{ij}}$ for treated individuals and $w_{ij}=\frac{1}{1-{\hat{e}}_{ij}}$ for control individuals, where ${\hat{e}}_{ij}$ denotes the estimated propensity score for the i^th^ household in the j^th^ administrative zone. The generalized linear mixed-effects model is used to estimate $e_{ij}$. The propensity score-based weights are applied to individuals in a sample to create a pseudo-sample balanced on the observed covariates between treatment groups.

*The optimal full matching (OFM):* with optimal matching, the treated individuals in the data set are matched with untreated individuals by minimizing the total distance between treated and untreated matched pairs (79). Unlike the matching methods, which may discard unmatched individuals, full matching forms matched sets that contain at least one treated and at least one control individual using the entire sample (80). The goodness of fit of these models can be checked by diagnostics of covariate balance (81), checking the balance of the weighted distribution of covariates in the two treatment groups. Once propensity scores have been estimated by Equation 2 and decisions have been made with respect to their utility, assessment in balance can be achieved by the calculation of the standardized difference for means (79).

#### 2.4.3 Multilevel model to estimate treatment effect

The final step of a propensity score analysis is the estimation of the average treatment effect (ATE) using a generalized linear mixed-effects model with the weights obtained from the propensity score method. To evaluate the treatment effect, the specification of a multilevel model for the outcome is


(3) $$g\;\left(E\;\left(y_{ij}\right)\right)=\gamma_{0}+\gamma_{1}t_{ij}+u_{oj},u_{0j}\sim N\left(0,\tau \right)$$

where $y_{ij}$ is the multidimensional poverty for rural household *i* ($i=1,2,\cdots ,n_{j}$) in cluster *j* (j=1,2,⋯,59 administrative zones), $\gamma_{0}$ represents the intercept, $\gamma_{1}$ represents the treatment effect, t represents the dummy indicator of crop commercialization of rural households, and $u_{0j}$ is the random intercept of zone *j* with variance τ.

Weights from propensity score methods can also be estimated using multilevel modeling techniques using sample weights (49, 82). The maximum likelihood estimation method for multilevel modeling (generalized linear mixed-effects model) with PS weights as sampling Weights allows for effectively handling multilevel data structures (47, 49, 77). Next, for multilevel modeling, we utilized R 4.0.2’s glmer function from the lme4 packages (83), which accepts precision weights but not sampling weights. Whereas precision weights are inverse variances, sampling weights are inverse probabilities of selection. Standard error estimates will vary, but treatment effect estimates will remain the same whether PS method weights are treated as sampling weights or precision weights. By employing weights from PS techniques, one hopes to prevent bias in parameter estimates and standard errors, much like sample weights do. However, one does not aim to increase the precision of the treatment effect (49, 82).

## 3 Results

### 3.1 Descriptive analysis

From the actual 3,239 rural households interviewed during the ESS 2018/19, we excluded households who were only administered for all agriculture data (post-planting, post-harvest, and livestock) or the household data or post-harvest agriculture data that was missing due to security problems (50). We also excluded missing values. Finally, in this study, 2,714 households living in rural areas of Ethiopia were analyzed. The descriptive analysis of rural household multidimensional poverty measures was estimated by the Alkire–Foster method to the ESS data of 2018/19. The multidimensional headcount ratio (H) and adjusted multidimensional headcount ratio ($M_{0}$) estimates are provided. The estimated incidence of poverty (multidimensional headcount ratio) demonstrated that 47.8% of the rural households of Ethiopia were multidimensionally poor. Moreover, the poverty rate considering the adjusted multidimensional headcount ratio is 20.1%. These poverty levels are lower than the study by Oxford Poverty and Human Development Initiative (OPHI) which indicated that the incidence of multidimensional poverty is higher in rural areas (55%) than in urban areas (16%) of Ethiopia in 2016 (15).

The multidimensional poverty of rural households is broken down into many household categories. The study estimates various multidimensional poverty indices using sixteen indicators, five dimensions, and the Alkire–Foster approach. Table 2 provides an overview of the multidimensional poverty dimensions and indicators for the full sample, illustrating the differences between households. It is important to note that explore which dimensions of poverty are deprived empirically in rural poor people of Ethiopia. The findings indicate that the living standard component is more deprivation-prone for the poor (26%), with nutrition and health coming in second (21.7%). These deprivations may be due to rising food prices. When it comes to the relative contributions of each dimension’s indicators, health is the most disadvantaged group (21.4%) for poor people, followed by risk exposure and coping mechanisms (13.5%). Our analysis result is in agreement with the study done by Amao et al. (84) and Mare et al. (20) found that the largest contributor to multidimensional poverty was living standards. According to another study, for the majority of the world’s regions, the multidimensional poverty index in rural areas is influenced more by the weighted living standards indicators than it is in urban areas (15). In Sub-Saharan Africa, where 5.9 percent (34.2 million) of the impoverished are accounted for, the standard of living is also the most prevalent deprivation profile (26).

**Table 2:** Summary of rural multidimensional poverty index (R-MPI) dimensions and indicators.

Dimension	Indicator	MPI	
Nutrition and health	Child malnutrition	0.002	0.217
	Health	0.214	
Education	Years of schooling	0.099	0.183
	School attendance	0.084	
Living standards	Electricity	0.054	0.260
	Improved sanitation	0.029	
	Drinking water	0.040	
	Housing	0.049	
	Cooking fuel	0.079	
	Assets	0.009	
Rural livelihoods and resources	Child labor	0.017	0.191
	Extension services	0.053	
	Agricultural assets adequacy	0.051	
	Social protection	0.070	
Risk	Denial of credit	0.015	0.149
	Exposure to risk & coping mechanisms	0.135	

Author’s computations made with the Ethiopian Socioeconomic Survey (ESS 2018/19).

In this investigation, the variation of multidimensional poverty for rural households by crop commercialization, gender, and region was presented. The comparison across these different groups shows the disparity of poverty. The results of rural multidimensional poverty index (RMPI) decomposition by treatment (crop commercialization) and gender group are presented in Table 3. The incidence of poverty is higher among completely subsistence households (53.8%) than among crop-commercialized households (39.4%) which imply that commercialized households are better off. The poverty rate for completely subsistence households (22.8%) regarding adjusted multidimensional headcount ratio is higher as compared to commercialized households (16.2%). In terms of the contribution of each domain, the commercialized households are less impoverished in terms of nutrition and health, living standards, and rural livelihoods & resources dimensions, while rural households are most deprived in the education and risk dimension, implying that crop commercialization improves household wellbeing.

**Table 3:** Rural multidimensional poverty index (R-MPI) dimensions and indicators decomposition by subgroups.

Dimension	Crop commercialization status		Gender		Total
	CS	CC	female	male	
**Indices by subgroups**					
H	0.538	0.394	0.512	0.467	0.478
Mo	0.228	0.162	0.217	0.195	0.201
Share of Population	0.584	0.416	0.256	0.744	1.000
**Each dimension’s contribution**					
Nutrition and health	0.219	0.211	0.219	0.216	0.217
Education	0.168	0.213	0.180	0.184	0.183
Standard of living	0.270	0.240	0.267	0.257	0.260
Rural livelihoods and resources	0.202	0.169	0.187	0.193	0.191
Risk	0.141	0.167	0.147	0.150	0.149

H, multidimensional headcount ratio; Mo, adjusted multidimensional headcount ratio; CS: completely subsistence; CC, crop commercialized.

Author’s computations made with the Ethiopian Socioeconomic Survey (ESS 2018/19).

Table 3 presents the rural MPI estimates using the latest ESS-2018/19 and indicates that the gender of the head of the household differences in poverty, female-led households are poorer than male-headed ones regarding the multidimensional headcount ratio. The findings indicate that compared to 46.7% of households headed by males, 51.2% of households headed by females are impoverished. Female-headed households (21.7%) are more deprived in terms of adjusted multidimensional headcount ratio than male-headed households (19.5%). The living standard component reveals greater disparities between families led by males and females in terms of each domain’s contributions. For the living standards dimension, female-headed households (26.7%) have a higher adjusted multidimensional headcount ratio ($M_{0})$ than male-headed households (25.7%). This implies that female-headed households contributed more to poverty. This finding is consistent with the previous studies by (85), which demonstrate that female-headed households had a higher probability of being poor as compared to male-headed households using multidimensional indicators of poverty. The female-headed households are significantly more likely to be poor compared to those in households headed by males in Ethiopia, South Sudan, and Sudan, while in Somalia, those living in male-headed households are more likely to be identified as multidimensional poor (86). Moreover, Covarrubias (87) in Mexico shows that the multidimensional poverty is greater for females than for males.

Table 4 demonstrates the deprivation profiles of poor people across ten administrative regions of the rural part of Ethiopia. The decomposition of rural MPI by region demonstrated that different regions had different levels of poverty. Examine the distributions of rural multidimensional poverty across different regions, with a particular focus on the multidimensional headcount ratio; the Afar area has the largest percentage of impoverished individuals (79.6%) and then the Somali (75.3%) and Gambela regions (62.8%), respectively. However, in the Amhara region, the incidence of poverty is the least (29.1%) next to Harar (29.5%) and Tigray (32.7%).

**Table 4:** Rural multidimensional poverty index (RMPI) breakdown according to regions.

Region	Indices according to subgroup (absolute)			Subgroups’ contribution to indices (%)		Each dimension’s contribution (%)				
	*H*	*M_0_*	proportion of *N*	*H*	*M_0_*	Nutrition & Health	Education	Standard of living	Rural livelihoods and resources	Risk
Tigray	0.327	0.129	0.136	0.093	0.088	0.193	0.224	0.213	0.204	0.166
Afar	0.796	0.345	0.099	0.166	0.171	0.233	0.131	0.296	0.223	0.117
Amhara	0.291	0.117	0.166	0.101	0.096	0.171	0.248	0.250	0.153	0.177
Oromia	0.455	0.190	0.136	0.129	0.129	0.226	0.232	0.264	0.126	0.152
Somali	0.753	0.324	0.107	0.169	0.173	0.225	0.121	0.284	0.227	0.143
Benisha.	0.366	0.156	0.052	0.040	0.041	0.226	0.190	0.221	0.165	0.199
SNNP	0.466	0.194	0.143	0.139	0.138	0.204	0.218	0.263	0.140	0.176
Gambela	0.628	0.270	0.066	0.087	0.089	0.228	0.152	0.256	0.278	0.086
Harar	0.295	0.113	0.049	0.030	0.027	0.241	0.268	0.170	0.161	0.161
Dire da.	0.484	0.210	0.045	0.045	0.047	0.230	0.160	0.218	0.201	0.191
Total	0.478	0.201	1.000	1.000	1.000	0.217	0.183	0.260	0.191	0.149

H, multidimensional headcount ratio; M_0_, adjusted multidimensional headcount ratio; N, Population.

Author’s computations made with the Ethiopian Socioeconomic Survey (ESS 2018/19).

Regarding the adjusted multidimensional headcount ratio ($M_{0}$), this regional $M_{0}$ disaggregation of this multidimensional approach indicated that Afar (34.5%), Somali (32.45%), and Gambela (27%) were the most impoverished regions, respectively. Regarding its role in the nation’s general poverty, the greatest percentage of poverty occurred in Somali (17.3%) next to Afar (17.1%), and SNNP (13.8%) regions. The findings align with the research conducted by (15); in 2016, the multidimensional poverty index was greatest in Somali and Afar regions (57 percent each) of Ethiopia. This could be because these rural household areas are primarily used for the production of livestock with fewer crop production systems. The recurrence of drought in Afar and Somali regions often aggravates the vulnerability of the mentioned main household livelihood activities (88).

### 3.2 Propensity score analysis

For the first step, we obtained a sample from ESS with 2,714 rural households nested in 59 administrative zones. The number of rural households per administrative zone was 8, 141, 46, and 30.02 at the lowest, highest, mean, and standard deviation, respectively. Out of the total sample of 2,714 rural households, the treatment group (crop commercialized) contains 1,129 (41.6%) observations, and the percentages of rural households implemented crop commercialization among the administrative zone values ranging from 0 to 3.43%; this indicated administrative zone-level treatment heterogeneity (Table 5). The sample of the ESS of 2018/19 was obtained with a two-stage stratified random sampling technique, but this study was only representative of the rural category of ESS data and not urban areas households. For this reason, sampling weights were not used in this investigation.

**Table 5:** Descriptive characteristics of rural households in this poverty study based on crop commercialization.

Label	The description of variables /measurement	Commercialized (*N* = 1,129; 41.6%)	Subsistence (*N* = 1,585; 58.4%)	*p*-value
Gender HH	Gender of the household head (=1, if male)	956 (84.67%)	1,064 (67.13%)	**<0.0001**
Education	Education of the household head (=1, if literate)	437 (38.71%)	480 (30.28%)	**<0.0001**
Currently married	=1, if Currently married	954 (84.50%)	1,131 (71.36%)	**<0.0001**
Married before	=1, if Widowed/divorced/separated	153 (13.55%)	383 (24.16%)	**<0.0001**
Participation Psnp	Participation in productive safety net program; =1, if the household received any assistance through PSNP in the past 12 months	64 (5.67%)	156 (9.84%)	**<0.0001**
Saving (yes)	Save in any way (at private & public banks, microfinance, SACCO, home, family, and equip) (=1, if yes)	300 (26.57%)	288 (18.17%)	**<0.0001**
Have account form fin	being a formal financial institution’s customer, i.e., private, public bank, and microfinance (=1, if yes)	285 (25.24%)	275 (17.35%)	**<0.0001**
Improved seed use	Household uses improved seed (=1, if yes)	357 (31.62%)	161 (10.16%)	**<0.0001**
Chemical fertusen	Households use chemical fertilizers on any one of your crop fields (=1, if yes)	481 (42.60%)	1,266 (79.87%)	**<0.0001**
Crop rotation	Household exercise crop rotation on your land holding (=1, if yes)	815 (72.18%)	474 (29.90%)	**<0.0001**
Age HH	age of the household head in years	45.95 (0.43)	44.23 (0.40)	**0.0036**
House hold size	the household size in number	5.11 (0.063)	4.65 (0.06)	**<0.0001**
Landsize	land size in hectares	1.07 (0.04)	0.43 (0.02)	**<0.0001**
Livestock holdings	In the tropical livestock unit (TLU)	3.58 (0.12)	4.04 (0.20)	**0.0492**
Remittance	Income transfers/gifts in birr	276.36 (51.38)	854.90 (121.27)	**<0.0001**
Share of non farm income	percentage share of total household cash income came from non-farm enterprises in the last 12 months	3.67 (0.41)	4.49 (0.41)	0.1555
Agri wage	The estimated yearly income from wage employment in agriculture during the preceding 12 months	228.92 (77.87)	331.56 (91.06)	0.3918
HHHRS agricultural	Total household hours for agricultural activities	58.74 (1.66)	44.61 (1.32)	**<0.0001**
Distance road	Household distance to the nearest major road in km	18.33 (0.54)	25.73 (0.88)	**<0.0001**
Distance market	Household distance to nearest market in km	58.40 (1.27)	88.40 (1.85)	**<0.0001**
Distance financial	Household distance to the nearest formal financial institution in km	19.68 (0.66)	40.22 (1.38)	**<0.0001**
Distance capitalre	Household distance to the capital of region residence in km	0.13 (0.003)	0.18 (0.003)	**<0.0001**
Income Livestock sales	Total income from livestock sales in Birr	3817.51 (363.91)	2952.89 (205.54)	**0.0387**

Bolded *p*-values are significant at the 1 and 5% level of significance; mean (standard deviation) is supplied for continuous, while frequency (%) is used to describe binary variables.

The choice of covariates is crucial for the analysis’s empirical sections. Regarding the propensity score model estimation, we examined the connections between crop commercialization and potentially confounding risk factors. The covariates were selected based on theory and prior empirical research to identify all predictors of crop commercialization status. The data-driven variables were chosen using the weighted *t*-test for a continuous covariate and the Rao-Scott chi-square test for categorical covariates. SURVEYFREQ and SURVEYMEANS procedures from SAS 9.4 were applied. The binary coding of the categorical covariates allowed for the reporting of the covariates’ estimated means and prevalence in Table 5. The findings show that there were statistically significant differences in 21 covariates (gender of household head, education of household head, currently married, married before (widowed/divorced/separated), participation in productive safety net program, saving, being a formal financial institution’s customer, use improved seed, use chemical fertilizer, exercise crop rotation, age of the household head, household size, land size, livestock holdings, remittance, total household hours for agricultural activities, distance to nearest major road, distance to the nearest market, distance to the capital of region residence, distance to the nearest formal financial institution, and total income from livestock sales in birr) out of 23 covariates between the crop commercialized and the complete subsistence groups of rural households. It means that the risk factors for the two groups differed systematically from one another.

For propensity score analysis, first, we identified 21 relevant covariates (Table 5), second, specify the generalized linear mixed model and estimate the propensity scores, and third, condition on propensity score techniques such as inverse probability of treatment weighting and optimal full matching. Fourth, we assessed covariate balance and found the failure of covariate balance might lead to re-estimating the propensity scores. So, based on Greifer and Thoemmes (89) recommendation, we repeat from second to fourth steps. Finally, we estimated propensity scores using a generalized linear mixed model of main effects by crop commercialization status on the selected 11 covariates identified as potential confounders (gender of head of the household, education of the head of the household, participation in productive safety net program, being a formal financial institution’s customer, use chemical fertilizer, exercise crop rotation, total household hours for agricultural activities, distance to nearest major road, distance to the nearest market, distance to the nearest formal financial institution, and total income from livestock sales in birr) of individual level and without cluster-level covariate, that is, random intercept model with lme4 package of R. Next, two sets of weights are established: one from the inverse probability of treatment weighting (IPTW) and the other from optimal full matching (OFM), using the predicted propensity score as a guide. These PS methods of weights across clusters help to reduce selection bias in the average treatment effect (ATE) estimates and evaluate covariate balance. The MatchIt package of R 4.0.2 was used to carry out the OFM (90). The advantages of optimal full matching (OFM) are that the matching order is not required to be specified and units do not need to be discarded. Unlike other distance matching methods, full matching can be used to estimate the ATE (90). The IPTW was performed with the WeightIt (91) package of R 4.0.2.

**Figure 1:**
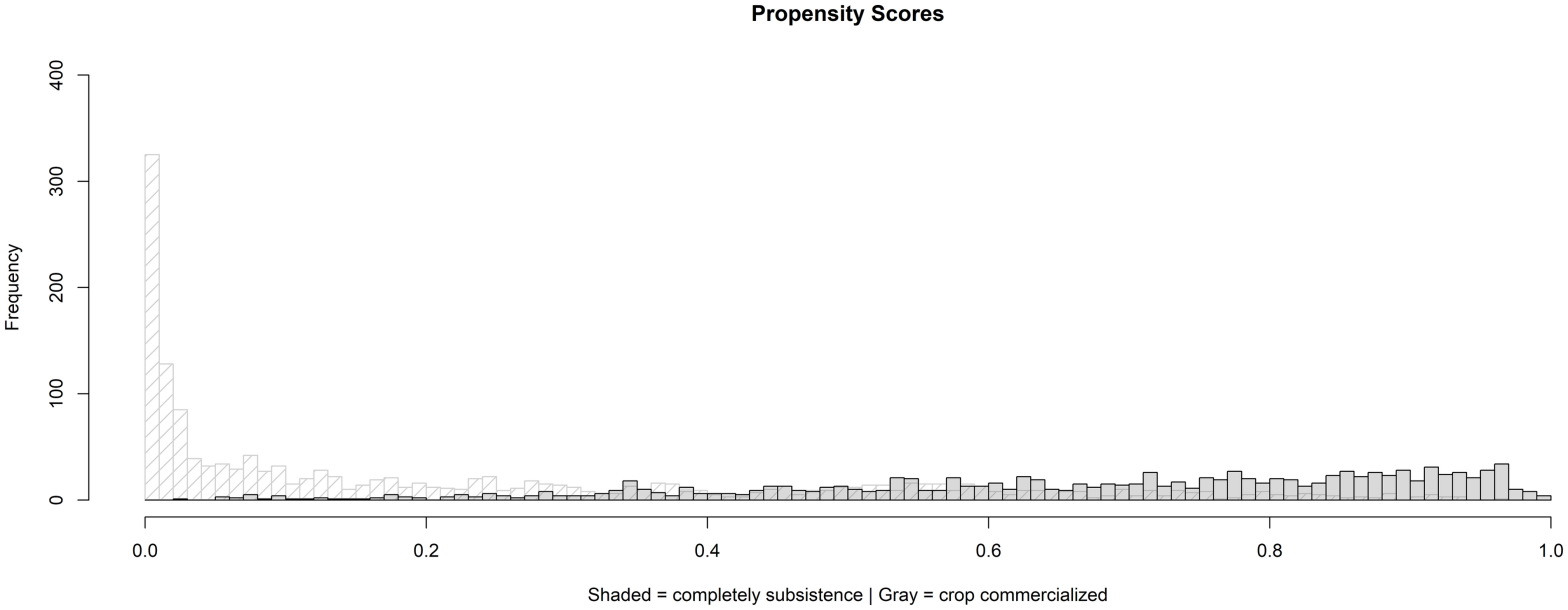
Common support using histograms of crop commercialization’s estimated propensity score using a random intercept model.

**Figure 2:**
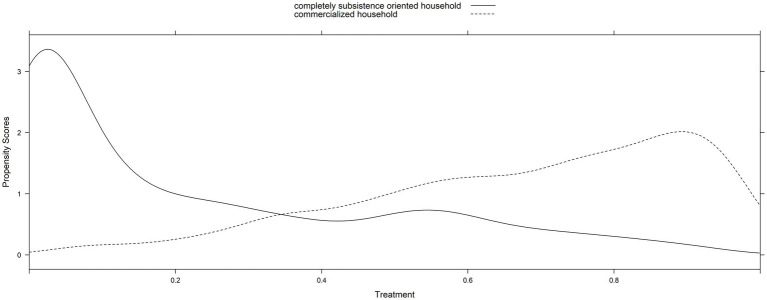
Common support using density plots of crop commercialization’s estimated propensity score using a random intercept model.

Examine the overlap assumption (the common support) in the crop commercialized and completely subsistence groups’ distribution to estimate the propensity scores that consider the data’s hierarchical structure. By comparing the crop commercialized and complete subsistence distributions using density plots and histograms, we assessed common support for the full sampled data and determined that it was sufficient (Figures 1, 2). The purpose of the PS adjustment is to produce study sampling data where the covariates are distributed similarly in the crop-commercialized and completely subsistence groups.

#### 3.2.1 Covariate balance assessment

To evaluate the covariate balance, we used the twang package (92) of R to get the weighted standard deviation for the complete sample as well as the weighted averages for each covariate in the crop commercialized and completely subsistence groups. The weighted absolute standardized difference was estimated. There is no clear consensus on what value of a standardized difference represents a key covariate balance between the sample participants who received treatment and those who did not. Several scholars have suggested that the absolute standardized mean difference (ASD) values ≤0.1 indicate that a covariate is adequately balanced between groups (93, 94). We achieved adequate covariate balance with IPTW; after the propensity score adjustment, the distributions for the two groups are nearly comparable; however, the ASD for two OFM variables was greater than 0.1. The results confirmed that the maximum absolute standardized mean difference was 0.154 for OFM (Table 6). These findings show that various PS techniques can yield varying degrees of covariate balancing when they use the same PS vector. The only PS approach that achieved appropriate covariate balancing was IPTW; thus, we moved on to the next phase using it.

**Table 6:** Covariate balance between crop commercialized and completely subsistence households for unadjusted, OFM, and IPTW samples.

Variables	Unadjusted			OFM			IPTW		
	CC	CS	ASD	CC	CS	ASD	CC	CS	ASD
Gender HH	0.847	0.671	**0.402**	0.847	0.859	**0.029**	0.782	0.742	**0.092**
Education	0.387	0.303	**0.178**	0.387	0.353	**0.072**	0.365	0.327	**0.078**
Participation Psnp	0.057	0.098	**0.153**	0.057	0.038	**0.069**	0.060	0.076	**0.058**
Have account form fin	0.252	0.174	**0.195**	0.252	0.230	**0.055**	0.240	0.207	**0.082**
Chemical fertusen	0.426	0.799	**0.778**	0.426	0.394	**0.065**	0.584	0.612	**0.057**
Crop rotation	0.722	0.299	**0.847**	0.722	0.799	**0.154**	0.549	0.507	**0.084**
HHHRS agricultural	0.152	−0.108	**0.260**	0.152	0.156	**0.004**	−0.010	0.000	**0.010**
Distance road	−0.146	0.104	**0.250**	−0.146	−0.183	**0.037**	−0.010	0.000	**0.010**
Distance market	−0.271	0.193	**0.464**	−0.271	−0.364	**0.093**	−0.027	0.000	**0.027**
Distance financial	−0.262	0.187	**0.449**	−0.262	−0.297	**0.035**	−0.001	0.000	**0.001**
Income Livestock sales	0.050	−0.036	**0.086**	0.050	−0.080	**0.130**	−0.021	0.000	**0.021**

CC, crop commercialized; CS, completely subsistence; ASD denotes the absolute value of the standardized mean difference; OFM, optimal full matching; IPTW, inverse probability of treatment weighting.

The logit and probit link functions of the generalized linear mixed model (GLMM) are compared using information criteria, and the logit link function of GLMM with lower Akaike information criterion (AIC) and Bayesian information criterion (BIC) values showed a better fitting model. Furthermore, the estimated variance explained by intercept differences in the zone (random effects) for the logit model (1.17) was larger than the probit model (0.42). The findings also suggested the existence of a statistically significant difference in the model with the random effect (zone) versus the one without it. Therefore, it is worth including the random effect for the zone because adding complexity to the model does improve it. Therefore, the GLMM with logit link was selected for analyzing crop commercialization’s impact on multidimensional rural poverty (Table 7).

**Table 7:** Model comparisons to evaluate random effect (administrative zones).

Models	Information criteria		Random effect variance (s)		
	AIC	BIC	$\tau_{00}\;(zone)$	ICC	*P*-value
Logit with GLMM	6457.3	6475.1	1.17	0.26	<0.001
Probit with GLMM	6457.5	6475.2	0.42	0.30	<0.001

In this study, we use the ESS 2018/19 data to estimate the ATE of households engaged in crop commercialization on rural multidimensional poverty by combining PS weighting with multilevel modeling. We estimated the ATE of crop commercialization on rural households’ multidimensional poverty applying a maximum likelihood (Laplace approximation) fit for a generalized linear mixed-effects model (GLMM) using the IPTW propensity score method as sampling weights. Next, we employed the R statistical software’s function glmer found in the lme4 packages (83).

In Table 8, we reported the estimates produced by the multilevel model with and without normalized weights. The treatment impact was expressed as the odds ratio of multidimensional poverty between rural households that were fully subsistence and those that were commercialized in terms of crops. The unweighted analysis’s estimations of the between administrative zone variance were lower than the normalized weights. Moreover, the ATE estimates without and with normalized weights indicate that rural households that were crop-commercialized showed a decline in being multidimensional poor compared to those households that were not crop-commercialized but had similar distributions of covariates. Though still statistically significant, the ATE estimates’ findings were less when weighted by IPTW than when they were not (Table 8). In addition, the results demonstrate that crop commercialization considerably (*p* < 0.01) lowered the likelihood of being poor, based on the weighted and unweighted treatment effects estimated. However, the unadjusted estimate of 0.75 (0.61–0.91) was less than the adjusted estimates, which raised the odds ratio. An odds ratio estimate of 0.79 (0.69–0.91) from the IPTW showed that rural households that commercialized their crops had a 21% lower probability of being poor than those that did not.

**Table 8:** Generalized linear mixed-effects models result in the impact of crop commercialization on rural households’ multidimensional poverty.

Fixed Effects	Unadjusted	Normalized weights
	Odds ratio (95% CI)	Odds ratio (95% CI)
Intercept	0.99 (0.76–1.29)	0.95 (0.71–1.28)
Cropcommstatus (treatment)	0.75 (0.61–0.91) ***	0.79 (0.69–0.91) ***
**Random effects**		
The variance of intercept: $\left(\tau_{00}\right)\text{zone}$	0.81	1.17
Intra-class correlation (ICC)	0.20	0.26

∗∗∗*p* < 0.01; ∗∗*p* < 0.05; ∗*p* < 0.1; *N* = 2,714, $N_{\text{zone}}=59$.

#### 3.2.2 Assessing model quality

For computing indices of model quality and goodness of fit, use measures of intra-class correlation coefficient (ICC). It measures the proportion of variation in multilevel or hierarchical data that is accounted for by a grouping (random) component, and it is computed by dividing the variation between groups by the overall variance (95). We estimated $ICC_{GLMM}$ from binomial generalized linear mixed-effects models (GLMMs) for binary data. The binomial distribution-specific variance $\delta_{d}^{2}$ (for example, $\pi^{2}/3\approx 3.29$ for binomial error distribution with the logit link function) for binary data (96, 97). In this study, for the binomial mixed models (logistic binomial GLMMs), the ICC value is 0.26. It is important to notice that ICC = 0.26, which is 26 percent of the total variance of rural multidimensional poverty, is due to administrative zones (random effects).

### 3.3 Crude prevalence and BLUP of multidimensional poverty of rural households

**Figure 3:**
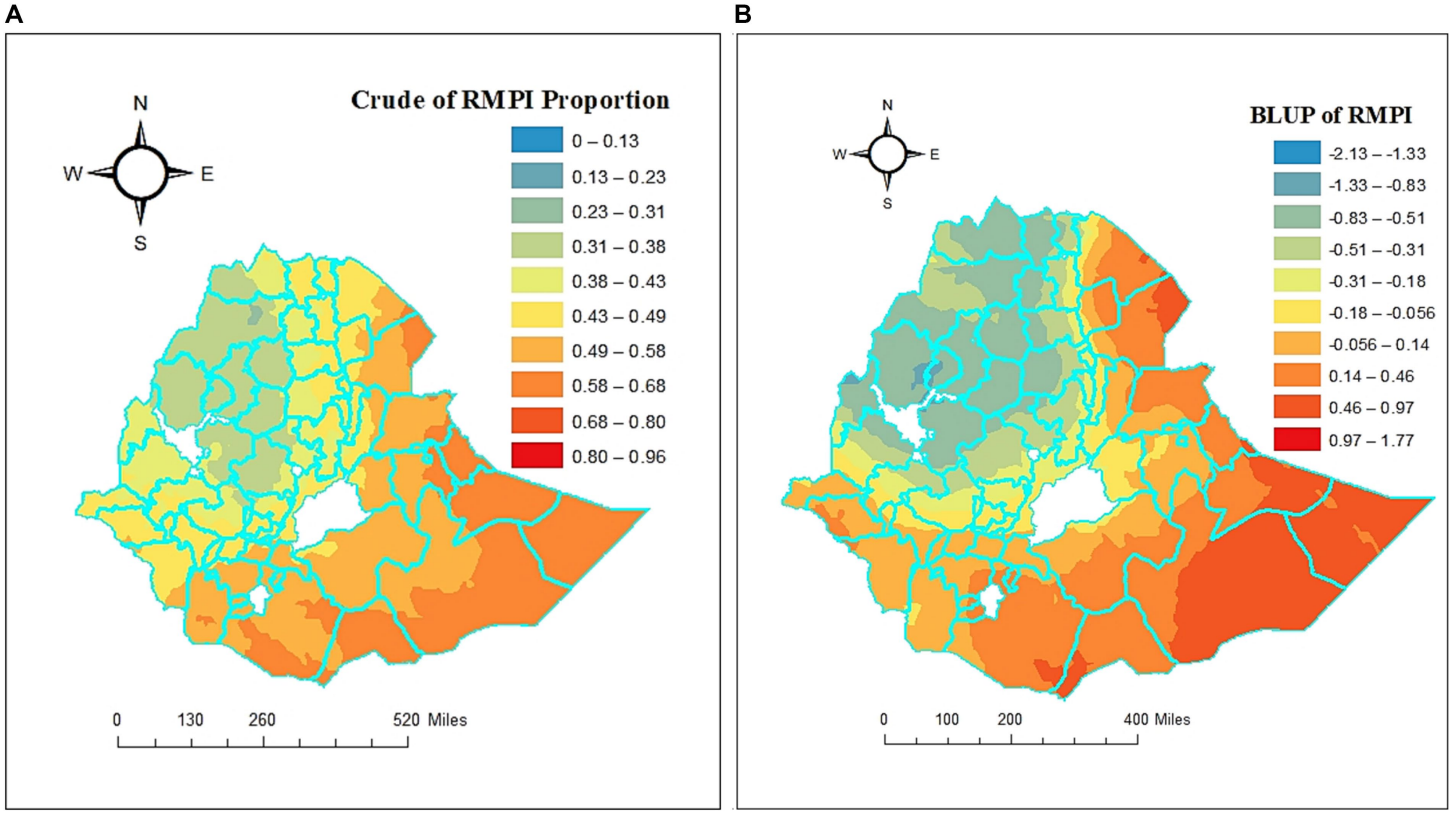
Crude and estimated BLUP prevalence regarding rural households’ multidimensional poverty in Ethiopian administrative zones: **(A)** Crude of RMPI proportion, **(B)** BLUP of RMPI.

Figure 3 presents the BLUP estimates and crude prevalence of poverty for rural households (RMPI) across 59 administrative zones of Ethiopia. The estimates of the BLUP and crude prevalence estimates for each zone were imported into ArcMap 10.7 and mapped. The results of empirical kriging were interpolated to the regions where data were not collected, and the resulting map was used to the prevalence of multidimensional poverty metrics in rural areas. The regions with the greatest and lowest crude prevalence of rural households experiencing multidimensional poverty were denoted by the colors red and blue, respectively (Figure 3). The maps of crude prevalence show that there were zonal disparities in rural multidimensional poverty in Ethiopia. The lowest incidence was noted in some parts of western Tigray; North and south Gonder, Awi, west Gojam, East Gojam in Amhara region; Metekel in Benshangul-Gumaz; some parts of west Shewa, some parts of East Wellega, some parts of Jimma, and some parts of North Shewa in Oromia region, while the highest prevalence of multidimensional poverty was observed in the Welwel & warder, some parts of Shebelle, Dege Habur, Jijiga, some parts of Shinile, some parts of Liben of Somali region; some parts of Borena in Oromia region; and some parts of zone 1 and zone 2 in Afar region in Ethiopia.

What we obtain from random effects are the conditional means given the observed data, which are the best linear unbiased predictions: BLUPs; random effects are random values rather than fixed parameters (98). Since the normal assumption typically shrinks these estimates toward zero, the results differ slightly from the fixed effects model. This research used GLMM to assess how different administrative zones perform on multidimensional poverty between Ethiopian rural households. A prediction of the true performance of each zone about the odds of being multidimensionally poor is shown in Figure 3. While a positive BLUP is linked to a greater likelihood of multidimensional poverty in the administrative zones, a negative BLUP is linked to a lower likelihood of multidimensional poverty in the administrative zones (99, 100). According to Figure 3, the best-performing administrative zone in terms of multidimensional poverty is the blue color, while the poorest-performing administrative zone is the red color.

The top five performing administrative zones were those with the lowest standardized BLUP values, and the top five worst-performing administrative zones were those with the highest standardized BLUP values concerning the likelihood of being multidimensional poor, according to the standardized BLUP estimates of each administrative zone (Figure 3). Accordingly, Awi, Oromia zone, North Wollo, and North Showa administrative zones across the region of Amhara, and Eastern Tigray administrative zone in regions of Tigray were the top best. The top poorest performing administrative zones in Ethiopia concerning the likelihood of being multidimensionally poor among rural households were Shebelle in the Somali region; Zones 2, 3, and 4 in the Afar region; and Konso special woreda in the SNNP region. In Ethiopian rural households, our article reveals that multidimensional poverty at administrative zonal levels varies significantly based on both crude and BLUP prevalence.

## 4 Discussion

We analyzed poverty in the measurement of a multidimensional approach using a representative sample of the latest Ethiopian socioeconomic survey data of rural households and provided evidence of the impact of crop commercialization on poverty. The Alkire–Foster method’s results for measuring multidimensional poverty indicate that there is a high level of multidimensional poverty in rural areas. A recent global multidimensional poverty index report indicates a high level of multidimensional poor population in rural Ethiopia (101). This study also showed living standard component is more deprivation-prone for the rural, multidimensional poor households of Ethiopia, with nutrition and health coming in second. Considering the relative contributions of each dimension’s indicators, health is the most disadvantaged group for multidimensional poor people of rural households, followed by risk exposure and coping mechanisms. This finding aligns with other studies in Kiribati and noted the high levels of deprivation in the living standards dimension of the entire rural population to multidimensional poverty (17). The study results in Ethiopia, Niger, and Nigeria also indicated that the living standards dimension contributes the most to the rural multidimensional poverty index (16). The previous study in Nigeria also confirmed that the living standard dimension of multidimensional poverty accounted for the largest share of deprivation suffered by rural women (102). The findings also showed that rural multidimensional poverty varied across crop commercialization, gender, administrative zones, and regions. The results also indicate gender differences in the experience of rural multidimensional poverty, showing that female-headed rural households are more affected by poverty than male-headed households. This finding is in line with Ichwara et al. (103) in Kenya and shows that the female-headed households have a higher probability of falling into poverty than male-headed households. Moreover, from the inter-household perspective in Brazil, female household heads are poorer than those in male-headed households. These results suggest that women are worse off than men in terms of employment, economic security, and access to resources (104).

We selected IPTW propensity score weighting methods to examine whether crop commercialization reduced the odds of being poor with the help of a generalized linear mixed-effects model. In this observational study, the findings show that rural households’ implemented crop commercialization significantly reduced the odds of being multidimensional poor. This result is consistent with the study done by Ogutu and Qaim (18) who remarked that commercialization significantly reduces multidimensional poverty headcount by 35% and has significant positive impacts on per capita income. It also showed that commercialization continuously reduces income-based poverty. Birhanu et al. (24) in teff-based mixed farming areas of Ethiopia also show cereal crop commercialization significantly reduces multidimensional poverty. The study conducted by Muricho et al. (65) in Kenya also found that agricultural commercialization significantly increases annual per capita household expenditure. In Rwanda and the Democratic Republic of Congo, the commercialization of bananas and legumes increases farm household incomes by 67% and contributes to improved household dietary diversity (41). The previous studies by the World Bank (34) have shown that poverty assessment studies within agriculture, the shift to cash crops, and the use of better seeds have reduced poverty. Specifically, the crop commercialized households have a diminishing impact on odds of being poorer due to the technology they have adopted as compared to their counterfactual group in reducing deprivations, i.e., improvements in welfare. The commercialization of farmers’ crops implies that higher agricultural yields can provide the necessary amount of revenue to lift people out of poverty. This supports the claim of (39, 105, 106) that the commercialization process helps to alleviate poverty among smallholders. Most of Ethiopian smallholders’ income comes from raising their livestock and crops rather than from outside the farm sources. Nevertheless, when compared to African nations, they do not make as much money from commercialization.

In this multilevel observational study, the estimated average treatment effect was lower with propensity score weights by IPTW than without the weighting approach. Moreover, by employing the maximum likelihood (Laplace approximation) fit of a generalized linear mixed-effects model with weights from propensity score techniques (IPTW), selection bias can be largely eliminated from the computed average treatment effect and its standard error. For clustered observational studies, propensity score methods taking account of the cluster structure reduce bias and are also necessary for valid standard error estimation. Previous studies have shown that the propensity score methods in multilevel observational studies reduce confounding bias compared to regression adjustment (107). Hence, incorporating unobserved cluster-level heterogeneity in propensity score and outcome analysis stages yields the least bias (47, 76). Several studies (45, 47, 48) have shown that the random effects model outperformed single-level models for propensity score estimation in reducing bias due to their ability to capture cluster-level heterogeneity. The results of this study demonstrated a considerable degree of spatial variability in rural multidimensional poverty both within and between Ethiopia’s administrative zones. The Ethiopian administrative zones’ varying climates could be the reason for this discrepancy. In Ethiopia, there was significant heterogeneity in rural multidimensional poverty at the administrative zonal levels, as indicated by the crude and BLUP prevalence. Using the standardized BLUP estimations of each administrative zone, the accomplishment of the administrative zones was measured regarding the likelihood of being multidimensional poor. The top best-performing administrative zones in reducing the chances of being multidimensional poor among rural households were found in the Amhara and Tigray regions. The administrative zones with the lowest performance rankings in reducing the likelihood of multidimensional poor across rural households in Ethiopia existed in regions of Somali, Afar, and Southern Nations, Nationalities, and Peoples (SNNP). This may be most of the time drought severely affects millions in the Somali, Afar Region, and southern parts of Ethiopia, and the majority of the rural poor in these regions (Afar, Somali) are pastoralists and agro-pastoralists who are dependent on livestock and farming for their survival and livelihood (35, 108). As a result, the BLUP ranking permits the lowest-performing zones with odds of being multidimensional poor to design appropriate interventions for a better way of moving out of poverty and enables the highest-performing administrative zones to recognize and enhance effective approaches.

### 4.1 Limitations of the study and areas for further research

The analysis of rural multidimensional poverty adds important implications for existing poverty literature and provides input to stakeholders for development policy design and for budget allocation targeted at overcoming rural multidimensional poverty in rural areas of Ethiopia. The study also provides limitations and suggestions on areas for future research. First, this study used the multidimensional poverty measure to obtain more direct insights into the experiences wherein rural households are deprived, accordingly, pointing to areas in which policy intervention should be directed. However, the availability of accurate and consistent data on specific areas is important for the effectiveness of the measurement. This may make results uncertain on the way toward better measurement. Hence, more work on advancements in the understanding and measurement of rural poverty with a multidimensional approach will be considered. Second, this study used the recent baseline Ethiopia socioeconomic survey 2018/19 (wave 4) data that revised the previous survey (wave 1, wave 2, and wave 3) instruments, module updates, and representative of all Ethiopian rural households. Hence, it may not be possible to compare rural multidimensional poverty across four waves without losing significant information. Third, this research unit of analysis was based on both the household level and administrative zone level; the results may vary by mediator variable. Thus, future research may use the household level, administrative zone level, and mediator variable unit of analysis that better reflects the real crop commercialization effect differences on multidimensional poverty reduction through mediator variables by incorporating administrative zone heterogeneity.

## 5 Conclusion

The majority of the earlier poverty research centered on the income or consumption expenditure approach. This study uses five dimensions with sixteen indicators to construct the multidimensional poverty index using Ethiopian Socioeconomic Survey data. This analysis also employs the Alkire–Foster counting technique for computing rural households’ multidimensional poverty and the distribution of poverty for various groups, such as crop commercialization, gender, administrative zone, and region. A generalized linear mixed-effects model with the propensity score technique (inverse probability treatment weighting) was used in the study to investigate the impact of crop commercialization on multidimensional poverty. This study’s empirical finding in rural multidimensional poverty of Ethiopia indicates that the living standard component is more deprivation-prone for the poor, with nutrition and health coming in second. The relative contributions of each dimension’s indicator to multidimensional poverty; health is the most disadvantaged group for poor people of rural households, followed by risk exposure and coping mechanisms. The findings also demonstrate that the BLUP estimates of multidimensional poverty vary substantially across Ethiopia’s administrative zones. Based on the results of this research, we infer that crop commercialization has a proactive impact on households’ likelihood of being multidimensional poor. Hence, the empirical result provides that increasing crop commercialization is the greatest important element directly accountable for the alleviation of Ethiopian rural areas experiencing multidimensional poverty. Addressing administrative zone variations of multidimensional poverty should also be a key component of poverty reduction strategies.

## Data Availability

The raw data supporting the conclusions of this article will be made available by the authors, without undue reservation.
